# Psychosocial wellbeing among children and adults with arthrogryposis: a scoping review

**DOI:** 10.1186/s12955-021-01896-5

**Published:** 2021-11-29

**Authors:** Sarah Cachecho, Jill Boruff, Trudy Wong, Francis Lacombe, Noemi Dahan-Oliel

**Affiliations:** 1 Shriners Hospital for Children-Canada, Montreal, Canada; 2 Schulich Library of Physical Sciences, Life Sciences, and Engineering, McGill University, Montreal, Canada; 3 Orthotist-Prosthetist and Patient Partner, St-Zotique, Canada; 4 Shriners Hospital for Children-Canada, School of Physical and Occupational Therapy, McGill University, Montreal, Canada

**Keywords:** Arthrogryposis multiplex congenita, Psychosocial wellbeing, Quality of life, Outcome measures

## Abstract

**Background:**

Arthrogryposis multiplex congenita (AMC) is a group of congenital conditions characterized by joint contractures in two or more body areas. Management of AMC starts early in life and focuses on improving mobility and function through intensive rehabilitation and surgical interventions. Psychosocial wellbeing is an important determinant of health and the psychosocial experience of individuals with AMC should be considered in the management of this condition. The aim of this scoping review was to explore what is known about the psychosocial wellbeing of children and adults with AMC, to identify the outcome measures used and to explore the factors associated with psychosocial outcomes in this population.

**Methods:**

A comprehensive search in four databases was conducted. Articles discussing psychosocial outcomes and outcome measures used with children or adults with AMC were included. Data on the measures used, psychosocial outcomes, and factors associated with psychosocial outcomes, were extracted and analyzed descriptively and synthesized narratively.

**Results:**

Seventeen articles were included in this scoping review, ten including the pediatric population, six including adults and one article including both children and adults with AMC. The most commonly used outcome measures were the PODCI in the pediatric studies, and the SF-36 in studies on adults. In the pediatric studies, psychosocial outcomes were often secondary, compared to the studies on adults. Results showed that in both children and adults, psychosocial outcomes are comparable with the levels of the general population. Qualitative studies reflected the affective needs of this population and issues with emotional wellbeing. Factors such as fatigue and pain were associated with poorer psychosocial outcomes in adults with an impact on social relationships, intimacy and family planning.

**Conclusion:**

Validated outcome measures, qualitative approaches and longitudinal studies are needed to better understand the psychosocial outcomes in AMC over time. Psychosocial support should be part of the multidisciplinary management of AMC throughout the lifespan.

**Supplementary Information:**

The online version contains supplementary material available at 10.1186/s12955-021-01896-5.

## Background

Arthrogryposis multiplex congenita (AMC) is a term that describes a group of congenital conditions characterized by joint contractures in two or more body areas [1] and affects 1 in 3000 live births [2, 3]. Joint contractures develop in-utero secondary to a decrease in fetal movement resulting in joint fibrosis and stiffness [4]. Contractures cause limited joint movement and muscle weakness in the involved body areas, and vary in distribution and severity. While contractures do not progress to previously unaffected joints, they may change over time due to growth and treatment. Depending on the severity and distribution of joint contractures, individuals with AMC may experience limitation in mobility and activities of daily living [5–7]. Other body systems such as the respiratory, gastro-intestinal, and central nervous system may be affected, depending on the underlying diagnosis [1, 3]. The management of AMC starts early in life with intensive rehabilitation, such as splinting and orthosis, range of motion exercises, strengthening programs, and surgical interventions to correct deformities, all of which aim to improve function [8–11].

The need to develop guidelines for the diagnosis, management and rehabilitation of AMC was identified during the Second International Symposium on Arthrogryposis in St-Petersburg in 2014 [12]. Promoting autonomy in daily activities, quality of life (QOL), and participation and integration in the community were emphasized [12]. In the qualitative study by Elfassy and colleagues, youth with AMC and their parents reported concerns in regards to affective wellbeing [13]. Medical and rehabilitation interventions focus mainly on the physical needs of individuals with AMC while psychosocial wellbeing is an important determinant of health and should not be overlooked [14]. Psychosocial wellbeing is a construct that involves social and emotional wellbeing, and refers to a positive mental state, such as feelings of happiness and satisfaction with life [15]. It includes psychological factors such as mental health, emotional wellbeing, and self-esteem, as well as interpersonal factors or the presence of positive relationships in one’s life [16]. Psychosocial wellbeing is one of the components of QOL that needs to be considered in populations with chronic conditions, such as AMC, and factors associated with better or poorer QOL need to be better understood [17]. Individuals with AMC may present varying degrees of physical limitations and may undergo several surgeries throughout their life [18]. Pain has been identified as an important issue and limiting factor in adults with AMC [19]. Therefore, in order to tailor interventions and optimize health and wellbeing throughout the lifespan, it is important to understand the psychosocial experience of individuals with AMC and to identify influencing factors.

The overall aim of this scoping review was to explore what is known about psychosocial outcomes of children and adults with AMC. Specifically, this scoping review aimed to (1) describe the psychosocial outcomes; (2) identify the outcome measures used to evaluate psychosocial outcomes; and (3) explore the factors that are associated with psychosocial outcomes among children and adults with AMC.

## Methods

A scoping review was selected to map the existing knowledge on the psychosocial outcomes in children and adults with AMC. A scoping review is designed to collect, evaluate and present a comprehensive map of existing evidence on a chosen research topic [20]. Arksey and O’Malley’s framework was used in addition to the recommendations by Levac, Colquhoun and O’Brien [21, 22]. The stages of this framework are: (1) identifying the research question, (2) identifying relevant studies, (3) selecting studies, (4) charting the data, and (5) collating, summarizing and reporting the results. An additional optional stage involves consultation with stakeholders. This scoping review was conducted by a team consisting of a young adult with arthrogryposis, clinicians (occupational therapist (OT) and social worker), an information scientist, and a clinician scientist. The research question (stage 1) is described above in the background. The PRISMA Extension for Scoping Reviews was used to verify that all aspects of the scoping review were considered [23].

### Identifying and selecting relevant studies

A health sciences librarian (J.B) developed the search strategy and performed the literature searches in MEDLINE, EMBASE, CINAHL, Cochrane Central, and Proquest Dissertations and Theses from database inception until March 6, 2020, with no limits to the period of time or language restrictions. The MEDLINE strategy was developed with input from the project team. After the initial MEDLINE strategy was finalized, it was adapted for use in the other databases. The search strategy (see Additional file 1) was designed to identify all relevant clinical literature on psychosocial wellbeing in AMC. Results from each database were exported into EndNote and duplicates were removed. Two independent reviewers (S.C and N.D-O) applied the selection criteria (Table 1) for titles and abstracts and then full texts, using the Rayyan software for screening of articles [24]. The two reviewers resolved any conflicts regarding inclusion of articles.

**Table 1:** Selection criteria

	Inclusion criteria	Exclusion criteria
Population age	Any age	None
Population diagnosis	Any type of AMC	Does not provide separate results for AMC in studies including other diagnoses
Study design	Quantitative, qualitative, mixed methods	Expert opinion and review articles
Language	English or French	Studies that did not provide English or French translation
Psychosocial outcomes	Describes psychosocial outcomes and uses an outcome measure with a psychosocial component	Does not describe psychosocial outcomes or use an outcome measure with a psychosocial component

### Charting the data

A data extraction form was created to identify study characteristics and key findings of included studies (i.e., study design, study purpose, sample size, outcome measure, intervention, and results). Two reviewers (S.C and N.D-O) independently piloted the data extraction form on three studies to ensure consistency in data extraction. Data samples were compared between the two reviewers and any discrepancies were resolved by discussion. A single reviewer (S.C) then extracted data from included studies and any uncertainties were discussed with the second reviewer. Level of evidence of included studies was assigned by two reviewers (S.C and N.D-O) using the Levels of Evidence for Primary Research guidelines by the Center for Evidence-based medicine [25]. In line with the scoping review methodology and the aims of our project, a critical appraisal and risk of bias assessment of included records was not performed.

### Collating, summarizing and reporting the results

The extracted data was synthesized according to three steps: (1) analyzing the data, (2) reporting the findings, (3) discussing the implications [21]. A descriptive numerical analysis and a narrative synthesis were used to analyze and report findings. The descriptive numerical analysis reflected the nature and distribution of the included studies. The primary units of analysis were the study purposes, the psychosocial outcome measures used and the psychosocial outcomes reported. The factors associated with psychosocial wellbeing from the included studies were synthesized narratively.

### Stakeholder consultation

Stakeholder engagement in research is important to ensure that research is relevant to the target population and that it answers questions that are of importance to the end-users [26]. Consulting with stakeholders may also help identify important themes to consider that are not necessarily reflected in the literature [21]. Exchanges on the elements to consider for the psychosocial wellbeing in this population were held during two virtual meetings in Spring and Summer 2020, with patient representatives (young adult with AMC, parent of a child with AMC and support group representative), clinicians (OT, physiotherapist) and researchers. The discussions that emerged from the stakeholder consultation were summarized and themes were reported inductively.

## Results

### Study selection

**Fig. 1:**
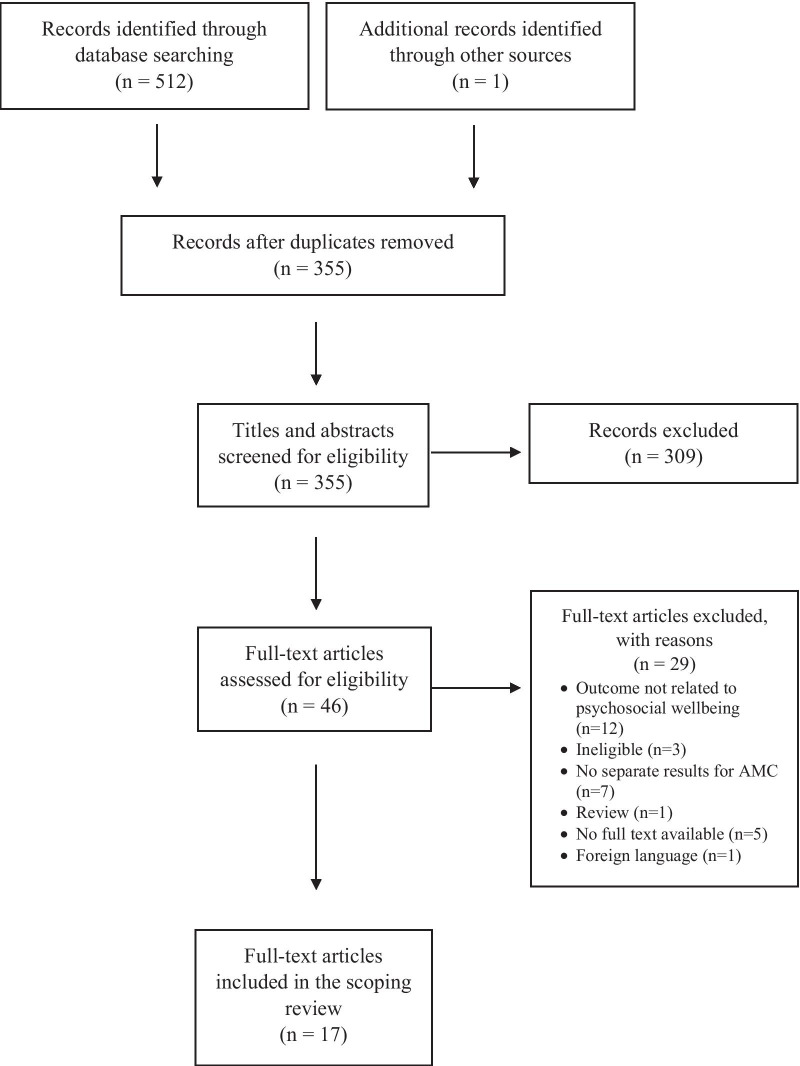
PRISMA flowchart

The search yielded 512 articles. After duplicates were removed, 354 abstracts were reviewed for study eligibility. Three hundred and nine articles were excluded at screening of titles and abstracts, and 29 articles were excluded at screening of full texts. One publication known to the authors that did not appear in the search was included as well. Therefore, 17 studies were included in the scoping review. Refer to Fig. 1 for PRISMA flowchart.

### Study characteristics and level of evidence

Included studies were published between 1997 and 2020, 15 of which were published after 2010. Studies were published across six different countries, with eight in the United States, five in Canada and the remaining in Europe. All studies were observational studies, specifically, cross-sectional studies (n = 7), retrospective cohort studies (n = 7), qualitative studies (n = 2) and one case series. Studies were classified as Level III (n = 6) or Level IV (n = 11).

### Sample characteristics

The 17 studies included 718 participants with AMC (range 1–177) ranging from infancy to older adulthood. Six studies included adult populations, 10 included pediatric populations, and one study included both children and adults with AMC. The pediatric studies population age ranged between 13 months to 20 years. Six of the 17 studies specified the underlying diagnosis (e.g., Amyoplasia, distal arthrogryposis) while the other studies identified participants as having “arthrogryposis” or “AMC”. One study had participants with different diagnoses but provided separate results for the participant with AMC. Refer to Table 2 for a full list of study and sample characteristics.

**Table 2:** Included studies characteristics and reported psychosocial outcomes

Study	Study design	Study purpose	Sample size, age	Psychosocial outcome measure	Psychosocial results
*Studies on children with AMC*					
Amor et al. [5]	Retrospective cohort study, Level III	To establish normative PODCI scores for children with Amyoplasia, and to determine whether the PODCI is sensitive to differing levels of severity of Amyoplasia and able to detect a change in the functional abilities of affected children over time	N = 74; range 2.2–18.1 years	PODCI (parent report)	Initial “happiness” score: N = 70; Mean ± SD = 81 ± 18, range 25–100 Comparison with normal population: *p* = 0.025* Change in “happiness” scores from initial PODCI administration to most recent PODCI administration (range: 5 months to 7.5 years) N = 39; Mean ± SD = 4 ± 21; * p* = 0.296 Change in “happiness” scores for patients who had UE surgery N = 10; Mean ± SD = 9 ± 20; * p* = 0.116
Cao et al. [27]	Retrospective cohort study, Level III	To review the mid to long term results of combined posterior capsular elbow release and triceps lengthening for elbow extension contractures in children with arthrogryposis; and to compare PODCI score with normal pediatric population and previously reported patients with arthrogryposis scores	N = 17 range at time of surgery 9.6 months–9.3 years; range at final follow-up 4–20 years	PODCI (parent and patient report)	Domain of “happiness” was similar to the population norms “Happiness” scores compared to the normal population Parent report for children < 11yo: N = 11, Mean ± SD = 81.8 ± 18.4; range 45–100; * p* = 0.4825 Parent report for children > 11yo N = 6, mean ± SD = 83.3 ± 29.6; range 25–100; * p* = 0.4958 Self-report (> 11yo) N = 6; mean ± SD = 77.5 ± 35.2; range 25–100; * p* = 0.2921
Church et al. [28]	Retrospective cohort study, Level III	To compare the effectiveness of the Ponseti method over a 5-year span in treating children with idiopathic clubfoot and clubfoot associated with arthrogryposis	Children with idiopathic clubfoot: N = 89, age 4.67 years ± 0.75 Children with AMC: N = 28; age 5.21 years ± 0.78	PODCI (parent report)	The children with idiopathic clubfoot and the children with AMC were statistically different in all 6 domains of the PODCI. The AMC group compared to the HC did not differ in the “happiness” domain “Happiness” scores: AMC group: mean 86 ± 21 Idiopathic group: mean 96 ± 11 AMC group compared to the idiopathic group: * p* = 0.029* AMC group compared HC: * p* = 0.75
Elfassy et al. [13]	Qualitative study, Level IV	To identify the needs surrounding rehabilitation as experienced by youth with AMC, caregivers and clinicians (OT, PT) and to propose solutions to develop family and client centered rehabilitation recommendations	Youth with AMC: N = 7, range 14–20 years; Caregivers: N = 11; range of children 2–12 years	Qualitative interviews	Two youth reported having affective needs such as anxiety and body image issues as well as being easily tired and having pain. Parents reported dealing with many aspects of their child's needs, including affective, physical and cognitive needs, pain, burden of care, mobility and adaptations. One parent mentioned how their child’s many medical appointments affected the parent’s ability to work
Eriksson et al. [31]	Cross-sectional study, Level IV	To describe HRQoL in a group of children with AMC and specifically to investigate whether there were any differences between children wearing orthoses compared to those not wearing orthoses	N = 33; range 5–17 years	CHQ-PF50, EQ-5D-Y	No difference found in the psychosocial summary score between the AMC group and the HC on the CHQ-PF50 Lower scores found in AMC compared to the HC for: “parent impact/emotional”, “self-esteem” and “behavior”. No differences found between the AMC sub-groups for these domains No difference between the AMC sub-groups on the EQ-5D-Y
Ho and Karol [6]	Retrospective cohort study, Level III	To determine the long-term ambulatory and functional outcome of patients treated for arthrogryposis with surgical knee releases	N = 32; range at time of knee release 0.8–6.8 years. Average length of follow-up 11.9 years, range, 2.2–23.6 years	PODCI (parent and patient report)	“Happiness” score (N = 22) within the average normalized scores (50 ± 10) Increased knee extension at final follow-up correlated positively with higher scores of “happiness”: * p* = 0.06; r = 0.49 BMI correlated negatively with “happiness” scores: * p* = 0.01 r = − 0.48
Pritchard-Wiart et al. [32]	Mixed method case series, Level IV	To describe modified toy car use by children with physical disabilities in home and community settings	Child with AMC and hypotonia N = 1; 13 months. Children with CP N = 4; range 22–58 months	Qualitative interviews, driving log	Results specific to child with AMC: Parents’ perception of child's motivation: average 9.5/10 Parents’ perception of child’s enjoyment: average 9.8/10 Parents reported the child was more curious, wanted to explore, and played more. Communication and motivation in general increased and he was able to keep up with his siblings and was more independent in his mobility
Spencer et al. [7]	Cross-sectional study, Level IV	To document the BMD of children with Amyoplasia and predominantly lower extremity arthrogryposis and compare it with age normative values. Secondarily, to compare bone mineral density with functional ability as quantified by the PODCI and the WeeFIM and with fracture risk in patients with arthrogryposis	N = 30; range 5–18 years	PODCI (unclear if patient or parent report)	The “happiness” normalized scores was within the norm 49
Wall et al. [29]	Retrospective cohort study, Level III	The hypothesis is that repositioning the hands, through distal humerus external rotation osteotomies, would allow for palm-to-palm grasp without arm cross-over, and would improve function and parent/patient satisfaction	N = 9; range 2–13 years; average follow-up 1.9 years; range 6 months–4 years	PODCI (parent report)	Post-operative mean «happiness» score: 89 (range 60–100)
Wall et al. [30]	Cross-sectional study (part of a larger longitudinal cohort study) Level IV	To investigate upper extremity functional and psychosocial measures in patients with arthrogryposis, comparing Amyoplasia and distal only involvement, utilizing the PROMIS and PODCI questionnaires	Amyoplasia N = 15; range, 6–10 years. Distal arthrogryposis N = 14; range 8–12 years	PODCI; PROMIS (UE function, pain interference, depression, anxiety, and peer relations)	Median “happiness” scores: All subjects (N = 29): 85 (70–95) Distal arthrogryposis (N = 14): 88 (80–99) Amyoplasia (N = 15): 85 (70–95) *p* = 0.40 Median PROMIS scores were in the normal range for both groups: Distal arthrogryposis: pain (46); depression (53); anxiety (51); peer relations (46) Amyoplasia: pain (50), depression (47), anxiety (49), and peer relations (56) “Peer relations” score was statistically lower for the distal arthrogryposis group
*Studies on adults with AMC*					
Altiok et al. [36]	Cross-sectional study, Level IV	To describe demographics and QOL, life satisfaction and functional mobility of young adults with AMC after transition from pediatric care	N = 23 range 18–36 years	SWLS, PROMIS-57 v2.0 profile, PROMIS Global health profile	56% were satisfied to extremely satisfied with life, and 22% were slightly dissatisfied to extremely dissatisfied. The five individuals dissatisfied with life also reported lower physical function, higher anxiety, depression and fatigue, and pain in multiple joints There was a moderate correlations between satisfaction with life with the PROMIS measures of global physical health (r = 0.559, * p* = 0.006), global mental health (r = 0.59, * p* = 0.003), and pain level (r = − 0.57, * p* = 0.005) 2/23 married, 1/23 engaged, 20/23 single; 7/23 lived independently, 12/23 lives with family, 3/23 lived in college, 1/23 lived in a nursing facility;
Dai et al. [37]	Retrospective cohort study, Level III	To analyze disability in a cohort of adults with AMC, according to the International Classification of Functioning, Disability and Health (ICF) by phenotype and genotype	N = 43; 33.2 ± 13.4 years	HADS	Chronic pain in 91% of the sample, with psychological consequences in half. The main psychological problems reported were: anxiety (43% with HADS > 10 for anxiety subscore; median 9 [6–19]), fatigue (34%), difficulty in sexual life (24%), altered self-esteem (17%), and feeling of solitude (15%). This affected work/study (17%) and planning of parenthood (7%) The prevalence of depression was low as per the HADS depression subscore median 3 (1–6) Results were similar for Amyoplasia group compared to other types of AMC All but one participant lived at home, and only one lived in a residence for disabled persons; 23% were students
Jones et al. [33]	Mixed methods cross-sectional study, Level IV	To validate the ODI as a pain and disability outcome measure in the AMC population	N = 50; range 21–85 years	ODI, SF-36, EQ-5D, in-house questionnaire	SF-36 score for MCS: 50.63 EQ-5D results showed less severe and/or disabling scores for pain and anxiety/depression When questions were phrased in an open-ended manner, no participant identified pain interference in the ODI domains of social activities, travelling or sex life. Subsequent closed-ended questions specific to the ODI domains showed significant pain-induced impairment in these domains
Nouraei et al. [34]	Cross-sectional study, Level IV	To examine the long-term functional outcomes of individuals with AMC with emphasis on the impact of their disability and treatment on their education, employment, and home life	N = 177; range 19–84 years	SF-36	SF-36: quality of life comparable to the general US population or better for several areas of health: “emotional” (77), “pain” (61), “social” (87) and “mental health” (74). The MCS (48) is comparable to the general US population (54) 45% were married, 27% were single and lived on their own, and 20% were single and lived with family or other support
Steen et al. [38]	Quantitative and qualitative study (Study 1: cross-sectional; Study 2: qualitative) Level IV	To describe body functions, activity and participation, and to explore psychosocial dimensions of adults with Amyoplasia	Study 1: N = 22; range 19–91 years Study 2 (sub-group of study 1): N = 8; range 20–60 years	Focus group interview	Most of the focus-group participants reported unwanted attention. The ambulatory group was more concerned with being different than the wheelchair-users, and were more preoccupied with what made them different. The ambulatory group talked a lot about feeling inferior, having to prove themselves and being tired, while the wheelchair-users did not talk about tiredness at all. In social interactions, the wheelchair-users emphasized one’s ability to influence interactions. The ambulatory group focused less on their own contribution to the interaction. Ten participants lived with a partner, and 5 had children
Sawatsky et al. [35]	Mixed methods cross-sectional, Level IV	To describe the relationship between surgically-managed joints and the QOL in adults with AMC	N = 83; 43 ± 12.5 years	SF-36, in-house interview	SF-36 domain scores were similar to a Canadian normative data-set for the psychosocial domains. SF-36 scores: Role limitations due emotional problems: 98 Emotional wellbeing: 86 Social functioning: 84 MCS score was average (49.5). There were no observable association between MCS and covariates Elbow surgery was positively correlated with a higher social functioning outcome Shoulder surgery was significantly inversely correlated with a higher social functioning outcome
*Studies on mixed pediatric and adult AMC population*					
Sodergard et al. [39]	Retrospective cohort study with qualitative component, Level IV	To describe EMG findings, disability and psychosocial outcomes in individuals with AMC	N = 52; average 16.3 years N = 27 older than 16yo	Semi-structured interview	Psychological evaluation showed that participants are able to obtain support from others, and have better mirroring and coping skills than most people. The mothers describe their children as kind, social and attractive but stubborn 14/27 lived independently, 5/27 lived in an adapted house, 8/27 had assistance. Many lived with their parents and only 2 married. Participants seemed to cope well socially and participated in social activities corresponding to their needs

AMC: Arthrogryposis multiplex congenita; BMD: bone mineral density; CHQ-PF50: Child Health Questionnaire—Parent Form 50; EQ-5D-Y: Euroquol five dimensions questionnaire-youth; HADS: Hospital Anxiety and Depression Scale; HC: healthy control; HRQoL: health related quality of life; MCS: mental component score; ODI: Oswestry Disability Index; OT: occupational therapist; PODCI: Pediatric Outcomes Data Collection Instrument; PROMIS: Patient-Reported Outcomes Measurement Information System; PT: physical therapist; QOL: quality of life; SD: standard deviation; SF-36: Medical outcome study short form 36; SWLS: Satisfaction with Life Scale

### Psychosocial outcome measures

The outcome measure that was most used in the pediatric studies was the Pediatric Outcome Data Collection instrument (PODCI) [5–7, 27–29]. The PODCI has six domains, one of which measures “happiness with physical condition”. All studies using the PODCI were parent-report and two studies also included patient-report among participants older than 11 years [6, 28]. One study used the Patient-Reported Outcomes Measurement Information System (PROMIS), more specifically the depression, anxiety, and peer relations domains [30]. Other outcome measures with questions pertaining to psychosocial wellbeing included the Euroquol five dimensions questionnaire for youth (EQ-5D-Y), and the Child Health Questionnaire–Parent Form (CHQ-PF50) [29]. Two studies used a semi-structured interview [13, 32].

The outcome measures used in the adult populations varied. Three studies used the Medical Outcome Study Short Form 36 (SF-36) [33–35]. Other outcomes measures used were the Satisfaction with Life Scale (SWLS) [38], the Hospital Anxiety and Depression Scales (HADS) [39], the Oswestry disability index (ODI), the EQ-5D [33], the PROMIS-57 v2.0 profile and PROMIS Global health profile [36]. One study used a focus group interview [40]. Two studies combined the use of a standardized outcome measures with a qualitative interview [33, 35]. The study with mixed pediatric and adult population used semi-structured interviews to evaluate psychosocial outcomes [39]. See Table 3 for a list of the outcomes measures used to evaluate psychosocial wellbeing in this population and their characteristics.

**Table 3:** Outcome measures for psychosocial wellbeing used in the included studies

Name of outcome measure	Purpose	Age group	Domains	Scoring	Cited in
Patient-Reported Outcomes Measurement Information System (PROMIS) 57 v2 profile	Evaluation of mental and physical health	Adults (> 18 years old)	Anxiety, depression, fatigue, pain interference, physical function, sleep disturbance, and ability to participate in social roles and activities as well as a single pain intensity item	The mean (T = 50) plus or minus one standard deviation (SD) is considered average (T = 40 to 60)	Altiok et al. [36]
PROMIS general health	Overall evaluation of one's physical and mental health	Adults (> 18 years old)	Physical and mental health	The mean (T = 50) plus or minus one standard deviation (SD) is considered average (T = 40 to 60)	Altiok et al. [36]
PROMIS pediatric	Evaluation of mental and physical health	Children (5–17 years old)	Depression, anxiety, peer relations	The mean (T = 50) plus or minus one standard deviation (SD) is considered average (T = 40 to 60)	Wall et al. [30]
Satisfaction with Life Scale (SWLS)	Measure of the judgmental component of subjective well-being	Adults (> 18 years old)	Satisfaction with life	7 point likert scale 31–35 Extremely satisfied 26–30 Satisfied 21–25 Slightly satisfied 20 Neutral 15–19 Slightly dissatisfied 10–14 Dissatisfied 5–9 Extremely dissatisfied	Altiok et al. [36]
Pediatric Outcomes Data Collection Instrument (PODCI)	To assess changes following pediatric orthopedic interventions for a broad range of diagnoses, with a focus on function and quality of life in children and adolescents	Children (2–18 years old)	1. Upper Extremity and Physical Function 2. Transfers and Basic Mobility 3. Sports and Physical Functioning 4. Pain/Comfort 5. Happiness with physical condition 6. Global Functioning	0–100 (better score = better health)	Amor et al. [5], Cao et al. [27], Church et al. [28], Spencer et al. [7], Wall et al. [29, 30]
Hospital Anxiety and Depression Scale (HADS)	Understand the experience of suffering in the setting of medical practice	Adults (> 18 years old)	Anxiety and depression	0–3 (lower = better), a score for each domain over than 11 indicates anxiety or depression	Dai et al. [37]
Child Health Questionnaire—Parent Form 50 (CHQ-PF50)	To assess health related quality of life for pediatric patients ages 5–12	Children (5–18 years old)	1. Physical Functioning 2. Role/social limitations 3. General Health perceptions 4. Bodily pain/discomfort 5. Family activity 6. Role/Social Limitations 7. Parent impact 8. Self-esteem 9. Mental Health 10. Behavior 11. Family Cohesion 12. Change in health	0–100 (higher score = better functioning)	Eriksson et al. [31]
Oswestry Disability Index (ODI)	Subjective percentage score of level of function (disability) in activities of daily living in those rehabilitating from low back pain	Adults (> 18 years old)	Pain intensity, Personal care Lifting Walking Sitting Standing Sleeping Sex life Social life Travelling	0–5 scale (lower = better) then percentage calculated based on number of answers 0–20%: minimal disability 21–40%: moderate disability 41–60%: severe disability 61–80%: crippling back pain 81–100%: bed bound or exaggerating	Jones et al. [33]
Euroquol five dimensions questionnaire: Adults version EQ-5D Youth version: EQ-5D-Y	To assess health relative quality of life. EQ-5D-Y	EQ-5D: > 18 years EQ-5D-Y: 4–18 years old	1. Mobility 2. Looking after myself 3. Doing usual activities 4. Having pain or discomfort 5. Feeling worried, sad or unhappy	Five levels of perceive problem for each domain: No problem, slight problem, moderate problem, severe problem, unable to extreme problem	Eriksson et al. [31], Jones et al. [33]
Medical outcome study short form 36 (SF-36)	A measure of health status	Adults (> 18 years old)	1. physical function 2. Role/physical 3. Role/emotional 4. Energy/fatigue 5. Emotional wellbeing 6. Social functioning 7. Pain 8. General health	0–100 (lower = better)	Jones et al., [33], Nouraei et al. [34], Sawatsky et al. [35]

### Psychosocial outcomes in children with AMC

Pediatric studies using the PODCI showed that “happiness” scores are generally high and comparable to the healthy population [5–7, 27–30]. These studies did not specifically aim to study psychosocial outcomes and most studies looked at outcomes of surgical interventions. One study comparing the effectiveness of the Ponseti method in treating idiopathic clubfoot and clubfoot associated with arthrogryposis showed that the arthrogryposis group had significantly lower “happiness” scores than the idiopathic group at follow-up, yet both groups reported high “happiness” scores [28]. Another study looking at long-term functional outcome of patients with arthrogryposis treated with surgical knee releases showed that the “happiness” normalized score was moderately positively correlated with the final average knee extension [6]. One study comparing functional and psychosocial outcomes between a group of children with Amyoplasia and a group of children with distal arthrogryposis showed no significant difference between the two groups in the “happiness” domain [30]. PROMIS scores of depression, anxiety, and peer relations domains were in the normal range for both groups but the “peer relations” score was statistically lower for the distal arthrogryposis group [30].

One study evaluating health related quality of life (HRQoL) in a group of children with AMC showed that, on the CHQ-50P, the AMC group scored lower than the healthy control on domains of parent impact/emotional, self-esteem and behavior which included satisfaction with school, athletic ability/outlook, looks/appearance, ability to get along with others and family, and life overall [31]. The same study compared children with AMC who wear orthoses to those who do not, and no significant differences were found between the two groups on the psychosocial outcome domains of the CHQ-50P and the EQ-5D.

In a qualitative study, youth reported, among other things, affective needs related to anxiety and body image and how those were impacted by factors such as pain and fatigue [13]. Parents reported the complexity of the needs to be taken into account when caring for a child with AMC, such as affective needs, physical needs, pain, mobility and adaptations, and how it affects the parent’s ability to work [13].

Finally, based on results from qualitative interviews, a study showed the benefits of early assistive mobility using a toy car on social and communication development, motivation, and curiosity [32].

### Psychosocial outcomes in adults with AMC

Three studies on adults with AMC used the SF-36 outcome measure and included 50 to 177 participants [33–35]. Findings showed that the mental capacity score of individuals with AMC, which encompasses the psychosocial domains of the measure, were comparable to the general USA and Canadian populations [33–35]. Separate scores for the psychosocial domains of the SF-36 were reported in two studies and were comparable to the general population [34, 35]. One of these studies described the relationship between surgically managed joints and the QOL in adults with AMC and showed that elbow surgery was positively correlated with a higher social functioning outcome and shoulder surgery was significantly inversely correlated with a higher social functioning outcome [35]. However, the strength of the correlation was not reported.

Altiok and colleagues reported that more than half of the adults with AMC in their study are satisfied to extremely satisfied with life [36]. The five participants who were dissatisfied with life also reported lower physical function, higher anxiety, depression and fatigue, and pain in multiple joints as per the PROMIS-57. Measures of global physical health, global mental health and pain level were moderately correlated with satisfaction with life. Scores of the PROMIS-57 and PROMIS global health psychosocial domains were consistent with the normative US population [36].

A study reported the presence of psychological problems associated with pain in less than half of the sample [37]. These problems were, in decreasing order of prevalence, anxiety, fatigue, difficulty with sexual life, altered self-esteem and feeling of solitude. They affected work and study in 17%, and planning of parenthood in 7% of participants. Only one person was reported to present with some depression as per the HADS. There was no difference in the psychosocial outcomes between the Amyoplasia group and other types of AMC [37].

Another study looking at pain and psychosocial outcomes in adults with AMC showed low levels of disabling scores for pain and anxiety/depression on the EQ-5D and no pain interference in the ODI domains on social activities or sex life [33]. However, closed-ended questions showed that pain led to significant impairment in social activities and sex life.

A focus group conducted with eight adults with AMC showed the different perceptions of individuals who are ambulatory and those who are wheelchair users, and the differences in how they handle stigma in social situations [38]. Participants who were ambulatory were more concerned about their appearance and about looking different; they expressed feeling inferior and focused less on their contribution in social interactions. On the contrary, wheelchair users were less concerned about their differences and focused more on how they can contribute to social interactions [38].

### Other factors affecting psychosocial outcomes

Five of the eight studies among adults with AMC reported data on the living situation and/or marital status of their participants [34, 36–39]. Two studies, one of which had a large sample size, reported a high number of individuals who were married or living with a partner (45%) [34, 38]. Other studies reported a lower marriage/partnership rate (< 13%) and had a younger sample [36, 37, 39]. Of those who were single, many lived with their family [36, 37, 39]. Positive relationships with family and friends, their support and involvement were reflected in three studies [32, 38, 39]. Support groups, mentioned in one study, were reported as beneficial as they provide the opportunity to meet, exchange and learn from others with similar conditions [38].

### Stakeholder consultation

Three themes emerged during the conference calls held with the stakeholders: the indirect cost of having a child with AMC, the importance of sports and leisure participation in quality of life, and the importance of youth empowerment. More specifically, the group discussed the indirect cost of caring for a child with AMC (e.g., many medical appointments, missed work days, travel time to appointments) and the impact on families, such as level of stress, interpersonal relationships among family members and dynamics within the family unit. Participation in leisure and sports were described as having a positive impact on the mental wellbeing of individuals with AMC. Promoting participation in activities such as adapted sports were considered as one of the goals when treating or caring for a child with AMC. Finally, providing youth with sufficient information and decision-making power regarding the treatments they receive (e.g. surgery) may lead to better psychosocial outcomes and better acceptance of their condition in the adult age.

## Discussion

The aim of this scoping review was to describe what is known about the psychosocial outcomes of children and adults with AMC. Overall, the mental health, emotional wellbeing and levels of happiness in children and adults with AMC was shown to be comparable to that of the general population.

### Psychosocial outcomes in children with AMC

Most pediatric studies aimed to evaluate surgical outcomes or compare health outcomes of children with AMC with the general population. Although most of the studies did not specifically evaluate psychosocial outcomes, they reported the level of “happiness” in children with AMC to be high and comparable to the general population when measured with the PODCI [5–7, 27–30]. One study using the CHQ-PF50 reported that children with AMC scored lower than the control group on parent impact/emotional, self-esteem and behavior [31]. Clinically important aspects of a condition may not be captured in generic QOL measures. A disease-specific QOL measure may be more sensitive to detect change following treatment and may be advisable to develop for AMC [40]. In pediatrics, when outcome measures include self-reported and proxy versions, both should be completed when possible as parents’ and children’s perspectives may differ [41, 42].

Studies evaluating QOL showed that individuals with physical disabilities do not report lower QOL, satisfaction with life or poorer perceptions of their wellbeing [43]. A systematic review on QOL in children with osteogenesis imperfecta (OI) showed that psychosocial QOL in this population is similar to the general population [44]. In a qualitative study, children with OI expressed emotions of fear, pain, isolation and being different [45]. Similarly, the qualitative study by Elfassy and colleagues described the perception of youth with AMC with regards to pain, fatigue, anxiety and body image [13]. Qualitative studies can be useful for consideration, in complementarity to quantitative studies, to understand stakeholder lived experiences.

While the topic of bullying was not mentioned in the articles of this scoping review, it may be worth exploring as well. Children with disabilities may be at higher risk of bullying [46] as described in a qualitative study with children and youth with cerebral palsy (CP) [47]. Participation in leisure activities, discussed during consultation with stakeholders, contributes to the mental and physical wellbeing of children with physical disabilities [48, 49]. However, there is a lack of studies looking at leisure and participation in children with AMC [50]. A case report described the positive effects of hydrotherapy on a child with AMC [41]. Although in this context the activity was therapeutic, it is a glimpse of the potential positive impact of a fun inclusive activity on a child’s self-esteem. The use of assistive technology, such as for mobility or self-care, may also facilitate social participation and interaction, and consequently improve self-esteem [32, 52, 53].

Family dynamics and challenges emerged as an important theme during the stakeholder consultation phase. A qualitative study described caregiver’s perspective on managing their child’s care and supporting their growth in different aspects of daily life [13]. These factors, and their impact on family dynamics, life balance and psychosocial wellbeing of the family as a unit should be considered in both research and in clinical practice.

### Psychosocial outcomes in adults with AMC

Although we identified fewer studies among adults than children with AMC, a greater number of the studies among the adult population addressed psychosocial outcomes specifically, such as QOL, satisfaction with life and mental health. Overall, the psychosocial outcomes among adults with AMC were similar to the general population and levels of depression were low [33–37]. Pain and fatigue were important factors that had an impact on QOL, mental health, social relationships and intimacy, as well as family planning [33, 36, 37]. Therefore, it is important to offer this population a multidisciplinary approach to address elements of pain and to provide psychological follow-up and support to promote positive outcomes later in life. Two of the studies reporting on the marital and living situation of adults with AMC had a high number of participants who were married or living with a partner [34, 38], which is similar to findings from other studies on adults with AMC [54–56]. Other studies reported a lower number of married participants, which may be due to their sample being younger [36, 37, 39]. Support with family planning and obstetrical consultation are important to address concerns related to parenthood [57].

From childhood to adulthood, individuals with AMC undergo an average of four to nine surgeries [18, 34]. A study looking at the relationships between surgically managed joints and QOL in adults found correlations between certain upper limb surgeries and social functioning [35]. Longitudinal studies could help evaluate the impact of surgery on functional and psychosocial outcomes, and determine the types of surgeries that should be recommended for this population.

## Limitations

Only French or English articles were included. Ongoing and non-published studies were not included in the search criteria. Stakeholders’ input at the stakeholder consultation stage of this scoping review involved informal discussions on psychosocial wellbeing and may have been optimized by using a more formal approach with specific guiding questions.

## Conclusion

Psychosocial outcomes among children and adults with AMC were found to be comparable to the general population. In adults, higher levels of pain and fatigue were associated with lower mental health and satisfaction with life and may have a negative impact on interpersonal relationships and intimacy. A large proportion of adults with AMC live with a partner or are married. The presence of meaningful relationships and access to support groups are valuable assets for this population. Psychosocial support should be part of the multidisciplinary management of AMC throughout the lifespan.

## Supplementary Information

**Additional file 1**. Ovid MEDLINE(R) ALL 1946 to March 06, 2020. Provides the search strategy used in Medline and applied to the other databases.

## Data Availability

Full data extraction table available upon request. Summary of results in Table 2.

## Abbreviations

AMC: Arthrogryposis multiplex congenita
CP: Cerebral palsy
CHQ-PF50: Child Health Questionnaire—Parent Form 50
EQ-5D-Y: Euroquol five dimensions questionnaire-youth
HADS: Hospital Anxiety and Depression Scale
HRQoL: Health related quality of life
ODI: Oswestry Disability Index
OI: Osteogenesis imperfecta
PODCI: Pediatric Outcomes Data Collection Instrument
PROMIS: Patient-Reported Outcomes Measurement Information System
QOL: Quality of life
SF-36: Medical outcome study short form 36
SWLS: Satisfaction with Life Scale
