# Supplementary material for: Psychosocial wellbeing among children and adults with arthrogryposis: a scoping review
Source: Health Qual Life Outcomes. 2021 Nov 29;19:263. doi: 10.1186/s12955-021-01896-5 (PMC8628374; doi:10.1186/s12955-021-01896-5)
Supplement: Supplementary file 1 — Additional file 1. Ovid MEDLINE(R) ALL 1946 to March 06, 2020. Provides the search strategy used in Medline and applied to the other databases. [file 12955_2021_1896_MOESM1_ESM.docx]

Additional file 1: Ovid MEDLINE(R) ALL 1946 to March 06, 2020

1 Arthrogryposis/ 1918

2 (Arthrogryposis or Amyoplasia or Multiple congenital contractures).tw,kf. 2118

3 1 or 2 2725

4 (Psychosocial or social or psychological or relationship* or friendship* or quality of life or behavior* or behaviour* or personality or attitude* or emotion* or affective or stress or depression or depressive or anxiety or anxious or coping or motivation or body image or sexual activity or wellbeing or well-being or mental health or self-esteem or psychiatr* or mood or moods or self-confidence or confidence or satisfaction or contentment or happiness or happy or sadness or dissatisfaction or unhapp* or PROMIS or PODCI or Weefim or SF36 or Eq-5D or patient reported or self report* or PROMs or PROM or Functional Independence Measure for Children or Paediatric Functional Independence Measure or Pediatric functional independence measure or WeeFunctional Independence Measure or Short Form 36 Health Survey or short form health survey or Pediatric Outcomes Data Collection Instrument).tw,kf. 4749159

5 "Quality of Life"/ or Psychosocial Support Systems/ or Depression/ or Stress, Psychological/ 393826

6 exp Family Relations/ 90904

7 Resilience, Psychological/ or Adaptation, Psychological/ or Stress, Psychological/ or Psychological Distress/ 196778

8 Attitude to Health/ or Attitude/ 129066

9 exp Emotions/ 234899

10 exp Anxiety/ 82674

11 motivation/ or achievement/ or aspirations, psychological/ 79688

12 body image/ or body dissatisfaction/ 17135

13 Sexual Behavior/ 55294

14 Mental Health/ 36662

15 Self Concept/ 55822

16 patient outcome assessment/ or patient reported outcome measures/ 9523

17 or/4-16 5000343

18 3 and 17 142
